# Tropical volcanic eruptions reduce vegetation net carbon uptake on the Qinghai–Tibet Plateau under background climate conditions

**DOI:** 10.3389/fpls.2023.1122959

**Published:** 2023-03-16

**Authors:** Zhiwei Yong, Zegen Wang, Junnan Xiong, Jie Tian

**Affiliations:** ^1^ School of Geoscience and Technology, Southwest Petroleum University, Chengdu, China; ^2^ School of Civil Engineering and Geomatics, Southwest Petroleum University, Chengdu, China; ^3^ Institute of Oil and Gas Spatial Information Engineering, Southwest Petroleum University, Chengdu, China

**Keywords:** vegetation net carbon uptake, background climate, volcanic eruptions, natural forcing, Qinghai–Tibet Plateau

## Abstract

The vegetation carbon uptake plays an important role in the terrestrial carbon cycle on the Qinghai–Tibet Plateau (QTP), while it is extremely sensitive to the impact of natural external forcings. Until now, there is limited knowledge on the spatial-temporal patterns of vegetation net carbon uptake (VNCU) after the force that caused by tropical volcanic eruptions. Here, we conducted an exhaustive reconstruction of VNCU on the QTP over the last millennium, and used a superposed epoch analysis to characterize the VNCU response of the QTP after the tropical volcanic eruptions. We then further investigated the divergent changes of VNCU response across different elevation gradients and vegetation types, and the impact of teleconnection forcing on VNCU after volcanic eruptions. Within a climatic background, we found that VNCU of the QTP tends to decrease after large volcanic eruptions, lasting until about 3 years, with a maximum decrease value occurring in the following 1 year. The spatial and temporal patterns of the VNCU were mainly driven by the post-eruption climate and moderated by the negative phase trends of El Niño-Southern Oscillation and the Atlantic multidecadal oscillation. In addition, elevation and vegetation types were undeniable driving forces associated with VNCU on QTP. Different water-heat conditions and vegetation types contributed to significant differences in the response and recovery processes of VNCU. Our results emphasized the response and recovery processes of VNCU to volcanic eruptions without the strong anthropogenic forcings, while the influence mechanisms of natural forcing on VNCU should receive more attention.

## 1 Introduction

Terrestrial ecosystems have significantly offset approximately 31% of global anthropogenic carbon dioxide (CO_2_) emissions and mitigated global warming over the past decades (IPCC, 2013; Fei et al., 2018). Both natural variability and anthropogenic factors have a significant impact on the vegetation net carbon uptake (VNCU) (Piao et al., 2013; Schimel et al., 2015; Zhang et al., 2018). Nevertheless, the superposition of natural and anthropogenic influences on the VNCU makes it challenging to distinguish between the two effects since the twentieth century. Therefore, it is necessary to investigate the causes of VNCU variation in a climate condition without strong anthropogenic influences, such as the background climate over the pre-industrial period.

During the period of 850-1850, the vegetation carbon uptake was dominated by internal variability in the terrestrial carbon cycle and its response to natural external forcings (volcanic eruptions and solar activity) (Zhang et al., 2019). In particular, large volcanic eruption events may have a significant effect on vegetation carbon uptake (Mercado et al., 2009; Zhang et al., 2019). Volcanic eruptions transform climate by injecting large amounts of sulfur dioxide (SO_2_) and sulfuretted hydrogen (H_2_S) into the stratosphere. They can scatter incoming solar radiation and absorb infrared radiation, cooling global surface temperatures (Robock, 2000; Toohey and Sigl, 2017). Such large-scale climate perturbations have the potential to disrupt terrestrial carbon uptake, which is sensitive to changes in climate (Segschneider et al., 2013). Previous studies have indicated that volcano-induced increases in precipitation also lead to increases in terrestrial primary productivity (Brovkin et al., 2010; Segschneider et al., 2013). Similarly, satellite observations and process-based ecosystem model simulations have identified global warming and increased spatial and temporal heterogeneity of precipitation as the main causes of the increase in NPP in recent decades (Piao et al., 2012; Cuo et al., 2021). But volcanoes studies using both modeled and observed in the twentieth century have indicated very divergent results for volcano-induced global precipitation responses, and one possible reason is that the observed volcanic eruption events are not sufficient for a robust statistical analysis (Iles et al., 2013; Maher et al., 2015; Tejedor et al., 2021). Many of these studies have focused on the past century, but only three major volcanic eruptions events occurred in the past half century (El Chichón in 1982, Pinatubo in 1991, and Hunga Tonga-Hunga Ha’apai in 2022), and both eruptions coincided with El Niño events (Rao et al., 2017). Thus, the response mechanisms of terrestrial carbon uptake to large volcanic eruptions in the background climate condition remain ambiguous.

Radiative forcing from volcanic eruptions can further influence climate patterns, such as El Niño–Southern Oscillation (ENSO) and the Atlantic multidecadal oscillation (AMO) (Tejedor et al., 2021), which can also have an impact on terrestrial carbon uptakes (Zhang et al., 2019). Numerous studies have investigated that interannual time scales of the terrestrial net biome production correlate with ENSO and the North Atlantic Oscillation (NAO), which affect the terrestrial carbon cycle (Bastos et al., 2016; Zhu et al., 2017; Zhang et al., 2018). These relationships are attributed to terrestrial temperature and precipitation controlled by decadal climate patterns, suggesting that climate patterns regulate short-term changes in terrestrial carbon uptake through temperature and precipitation (Zeng et al., 2005). Although the effect of climate patterns on the VNCU has been explored, the spatial and temporal representativeness of VNCU and the response process after large volcanic eruptions are unclear. Therefore, more researches are imminently required to comprehensively advance the understanding of VNCU responses to natural external forcings, which will contribute to regional carbon management to mitigate climate change.

As the largest and highest plateau on Earth, the atmospheric circulation and geographical features of the Qinghai–Tibet Plateau (QTP) are critical to the global climate system (Jiang et al., 2014; Yu et al., 2022). The unique climate over the QTP is mainly affected by its geographical location, altitude, and monsoon. In addition, the ecosystems on the QTP are extremely sensitive to warming climate. Numerous studies on the QTP have focused on the response of terrestrial ecosystem productivity to climate change during the 1980s (Ye et al., 2020; Sun et al., 2022a; Sun et al., 2022b). However, the terrestrial ecosystem productivity shows significant effects of natural variability and anthropogenic forcing, but the superposition of natural and anthropogenic influences on terrestrial carbon cycling during this period makes it difficult to sort out both effects (Lehner et al., 2015). Thus, the last pre-industrial millennium (850-1849) constitutes a test period for characterizing the VNCU and their variation mechanisms without the complications of anthropogenic influences (Zhang et al., 2018). This helps us to sufficiently investigate the spatial and temporal processes of VNCU in response to volcanic eruptions. Furthermore, plant traits and elevational gradients are also undeniable factors related to VNCU on the QTP (Gao et al., 2019; Ye et al., 2020; Sun et al., 2022b). Paying attention to the effects of volcanic eruptions on VNCU across elevational gradients and vegetation type contributes to our understanding of carbon cycle responses to natural forcing.

Here, we reconstructed the net primary production (NPP) based on earth system models (the Community Earth System Model Last Millennium Ensemble (CESM- LME) and the Meteorological Research Institute Earth System Model version 2.0 (MRI-ESM2-0) model) over the last millennium. We investigate the response and the recovery process of VNCU on the QTP to volcanic eruptions over the last millennium using a superposed epoch analysis (SEA), and the updated volcanic forcing data comes from the suite of ice core records from Greenland and Antarctica. Further, we will characterize the different responses of vegetation net carbon uptake to volcanic eruptions across elevation gradients and vegetation types. Finally, we extensively evaluate the impact of tele-connections forcings on NPP anomalies after volcanic eruptions.

## 2 Materials and methods

### 2.1 Climate model

Monthly values of climate variables are obtained for the last millennium (850–1849 CE) simulations from the MRI-ESM2-0 model participating in the fourth phase of the paleoclimate modeling intercomparison project (PMIP4) (Jungclaus et al., 2017; Kageyama et al., 2018; Yukimoto et al., 2019), which is conducted as part of the Coupled Model Intercomparison Project Phase 6 (CMIP6). The MRI-ESM2-0 model is developed by the Meteorological Research Institute of Japan, and covers a preindustrial control experiment from 850 to 1849 CE. The control experiments of the MRI-ESM2-0 model are driven based on observations forcing, and followed the protocol specified by CMIP6 (Yukimoto et al., 2019). Our analysis is a comparison between the MRI-ESM2-0 model and an atmosphere-ocean climate model (CESM-LME simulation number 10) (Otto-Bliesner et al., 2016), ensuring that the differences in the estimated responses are the result of the proxy information from climate model. CESM is improved by the Community Earth System Model Paleoclimate Working Group at the National Center for Atmospheric Research. CESM-LME provides a better understanding of the proxy record and climate variability since 850 CE (Otto-Bliesner et al., 2016).

Since uncertainty about reconstruction increases significantly before 1000 CE, we restrict our study to the period 1000 to 1850 CE. We employ the annual mean of the reconstructed near-surface air temperature and the precipitation from MRI-ESM2-0 model output. both climate variables are reconstructed on approximately a 1°grid. We select the same MRI-ESM2-0 variables in the CESM-LME and perform the equivalent analysis. However, the spatial resolution of CESM-LME is approximately 1.9°×2.5° grid. To summarize the multi-model ensemble statistics, we employ bilinear interpolation to regrid the CESM-LME and MRI-ESM2-0 model to a common 1°×1° grid, and then take the multi-model median for each grid cell (Bevacqua et al., 2022). Because medians are more robust to outliers from the individual model than the mean values (Li et al., 2021). Multi-model median is also used to reconstruct the NPP over the last millennium (Multi-model median).

### 2.2 NPP reconstructed

To quantify the net amount of VNCU on the QTP, we reconstructed the net primary production (NPP) over the last millennium based on three climate models as the level of VNCU. Model NPP is computed using the Miami model for annual average temperature, annual total precipitation. Based on relationships between annual average temperature (AVT), annual total precipitation (ATP), and NPP. Miami model followed the Walter ratio, it has been observed to increase NPP in arid regions by 1.0 gCm^-2^ for every mm precipitation. Based on the Van’t Hoff rule, The temperature function represents a doubling of NPP every 10°C between 10°C and 20°C (Lieth, 1973; Zaks et al., 2007; Sha et al., 2020) as follows (Adams et al., 2004):


(1) $$NPP=MIN\left\{\left(\frac{3000}{1+EXP\left(1.315-0.119T\right)}\right),\left(3000\left[1-EXP\left(-0.000664P\right)\right]\right)\right\}$$

Where, *T* is the AVT (°C), *P* is the ATP (mm), and the *NPP* to a carbon unit (gCm^-2^year^-1^). Additionally, we acquired the MOD17A3HGF NPP data for 2000, 2005, 2010, 2015, and 2020, with a spatial resolution is 250m×250m grid. Here, MOD17A3HGF NPP data were used as a comparison for the model NPP reconstruction, and were used to verify the robustness of the model NPP reconstruction (Sun et al., 2022a). Specifically, we calculated the spatial distribution of the annual mean of the MOD17A3HGF NPP data, and compared it with the spatial distribution of the annual mean of the model NPP reconstructed from the three climate models. Finally, visualized the root mean square error (RMSE), spatial correlation coefficient, and standard deviation between the four datasets using Taylor diagrams (Taylor, 2001).

### 2.3 Volcanic forcing index

The global volcanic forcing reconstruction (named “eVolv2k_v2”) dataset is from a suite of ice core records from Greenland and Antarctica. the eVolv2k_v2 dataset includes reconstructions of the magnitudes of major volcanic stratospheric sulfur injection (VSSI) events and their approximate source latitudes from 500 BCE to 1900 CE. This data significantly improves the dating and synchronization of ice records and refines the methods used to derive VSSI estimates to obtain an accurate description of the large volcanic eruption events (Toohey and Sigl, 2017; Tejedor et al., 2021). We follow the previous study, and employ those volcanic eruption events that occurred in the tropics (from 25° N to 25° S) with magnitudes greater than Pinatubo volcanic event (VSSI > 8.78 Tg S) during the period of 1000 CE to 1850 CE (Tejedor et al., 2021). We select these volcanic eruptions events occurred in the tropics regions because the climatic signal from volcanic eruptions is associated with seasonal changes in the Intertropical Convergence Zone (ITCZ), which promotes aerosol transmission between hemispheres (Kravitz and Robock, 2011). Then, to avoid the potential bias caused by “double events”, when two events occur within 10 years of each other, we choose only the second event. We finally obtain 13 volcanic eruption events to analyze their influence on the VNCU of the QTP, whose detailed descriptions are summarized in **Table S1**.

### 2.4 Climate indices

These climate indices this study used come from the Paleo Hydrodynamics Data Assimilation product (PHYDA), including a global reconstruction of hydroclimate and associated dynamic climate variables over the past 2000 years (Steiger et al., 2018). PHYDA is conducted to reconstruct hydroclimate based on a data assimilation approach, which optimally combines 2,978 paleoclimate proxy data time series with physical constraints from atmosphere-ocean climate models. Here, we use several PHYDA-reconstructed dynamical variables: the monthly Niño 3.4 SST indices (Niño 3.4 indices), AMO, and the location (in the degree of latitude) of South Asia ITCZ (ITCZ_SA). These climate indices are employed to evaluate whether volcanic forcing induced variations in NPP could be modulated by teleconnection patterns (Rao et al., 2017).

### 2.5 Vegetation distribution map and DEM

The vegetation distribution map of the QTP is obtained from the 1:1000000 Chinese vegetation map in the Resource and Environment Data Cloud Platform, and the spatial patterns of the main vegetation types (alpine meadows, alpine grasslands, and forests) on QTP are shown in **Figure S1**. Digital elevation model (DEM) product is SRTMDEM data acquired from Geospatial Data Cloud, the spatial resolution of SRTMDEM is approximately 90m×90m grid. Before the analysis, the DEM data was resampled to 1°×1° grid to make it consistent with the spatial resolution of the model output.

### 2.6 Analysis methods

The impacts of tropical volcanic eruption events on the VNCU are detected and quantified using SEA. SEA has been widely used for climate response to volcanic eruption events (Stoffel et al., 2015; Tejedor et al., 2021). However, one assumption of SEA is that the year of the volcanic eruption event is accurate (**Table S1**) (Rao et al., 2017). With regard to each volcanic event, the year of volcanic eruption is designated as year 0, and the NPP, AVT, and ATP values of model output were extracted for the 3 years before and 5 years after the eruption to obtain the SEA matrix. Before averaging the responses of the NPP, AVT, and ATP for all 13 events, anomalies relative to the reference period (averaged over 3 to 1 years before the volcanic eruption events) were calculated to obtain a composite of all events over the 5 years after the volcanic eruption. We chose a short reference period of 3 years to avoid the influence of the average background condition due to low-frequency changes over the last millennium. Next, we tested the statistical significance of the SEA results with a two-tailed t-test.

According to previous studies, the volcano-forced VNCU anomalies in the SEA analysis can be modulated by the teleconnection model (Rao et al., 2017). Hence, we estimate spatial field correlations (Pearson correlation) between three teleconnection patterns and both VNCU variations of the QTP under the impact of specific volcanic eruption events. It is worth noting that the temporal correlations are considered on linear detrended data. Here, three indices (Niño 3.4 indices, AMO, ITCZ_SAM) are selected to examine the teleconnection patterns. Niño 3.4 indices and AMO indicated ENSO influence of the tropical Pacific and the SST anomalies of the North Atlantic, respectively. ITCZ_SAM shows the location (in the degree of latitude) variation of South Asia ITCZ.

The magnitude of VNCU in response to volcanic eruption events varied among vegetation types. Therefore, we analyze the dynamics of VNCU anomalies after the specific volcanic eruption events in each of the three typical vegetation types. Furthermore, vegetation activity often displays a clear elevational pattern (Körner, 2007; Gao et al., 2019), where elevation-dependent temperature changes are considered to be the main reason for the differences in vegetation activity along the elevation gradient (Li et al., 2016). Hence, we divide the elevation of QTP into 1000m intervals and then evaluate the elevation dependence of VNCU in response to volcanic activity.

## 3 Results

### 3.1 Evaluation of NPP model output

**Figure 1:**
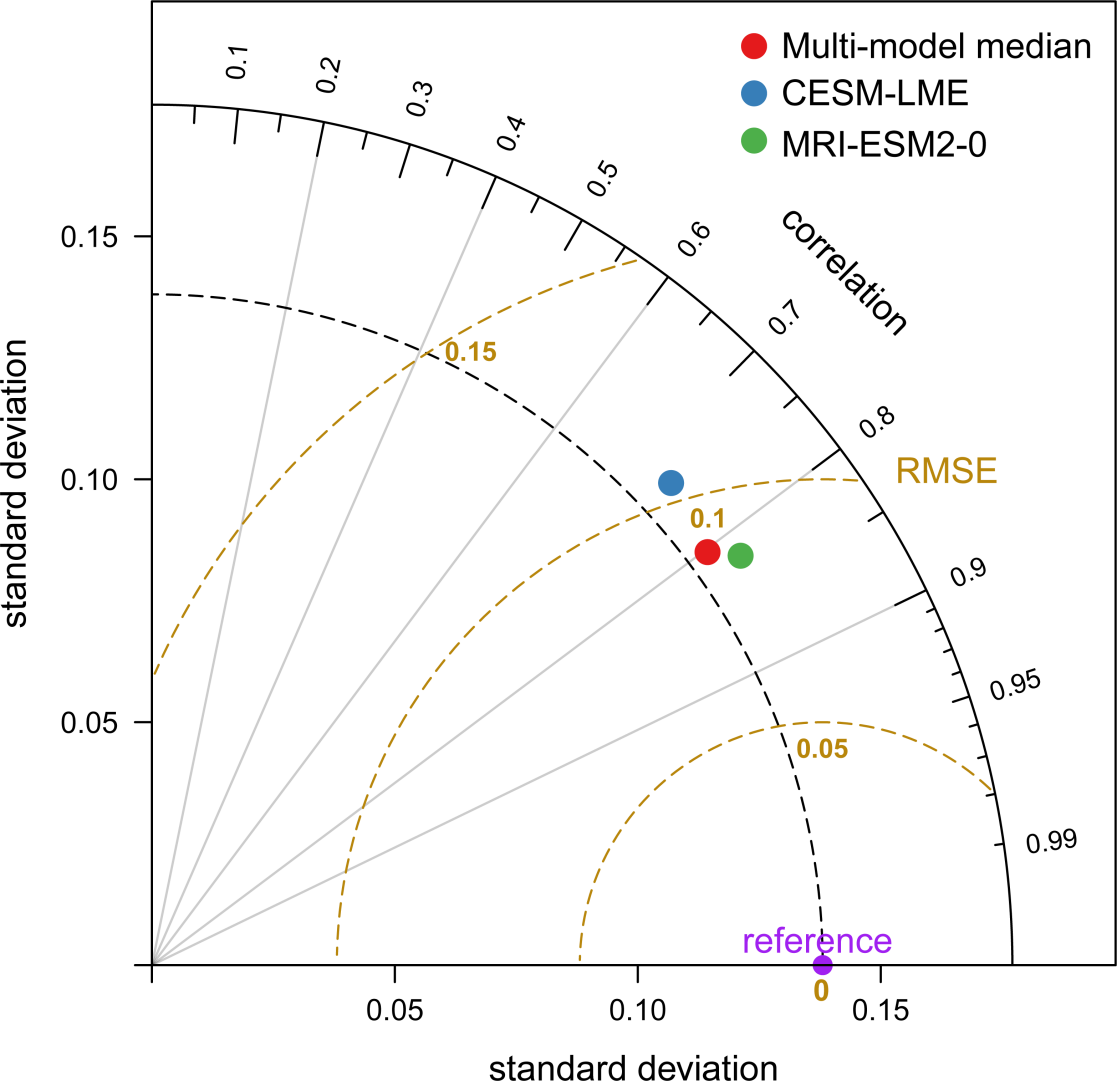
Taylor diagram displaying pattern statistics of the net primary production (NPP) reconstructed over the last millennium between three climate models and the MOD17A3HGF NPP data. MOD17A3HGF NPP data is considered as the reference. The radial distance from the origin indicates the standard deviation of the model. The spatial correlation coefficient between the model and the observation is shown by the azimuthal position. The root means square error between the model and the reference is displayed by the distance between them.

The Taylor diagrams are employed to measure the similarity between reconstructed and observations because it provides statistical information such as the RMSE, spatial correlation coefficient, and standard deviation (Taylor, 2001). Here, the Taylor diagram was used to examine the performance of the NPP model outputs on the QTP (**Figure 1**), and the annual mean of three models and MOD17A3HGF NPP data were normalized before analysis. Compared to the MOD17A3HGF NPP data, the spatial correlation coefficients in NPP reconstructed range from 0.70 (CESM-LME) to 0.81 (MRI-ESM2-0). The RMSE between model results and MOD17A3HGF NPP is approximately 0.1. The standard deviations of all model NPP reconstructed are approximately 0.14. These results show that three models have high abilities in capturing the spatial patterns of the NPP.

### 3.2 NPP response to tropical volcanic eruption

**Figure 2:**
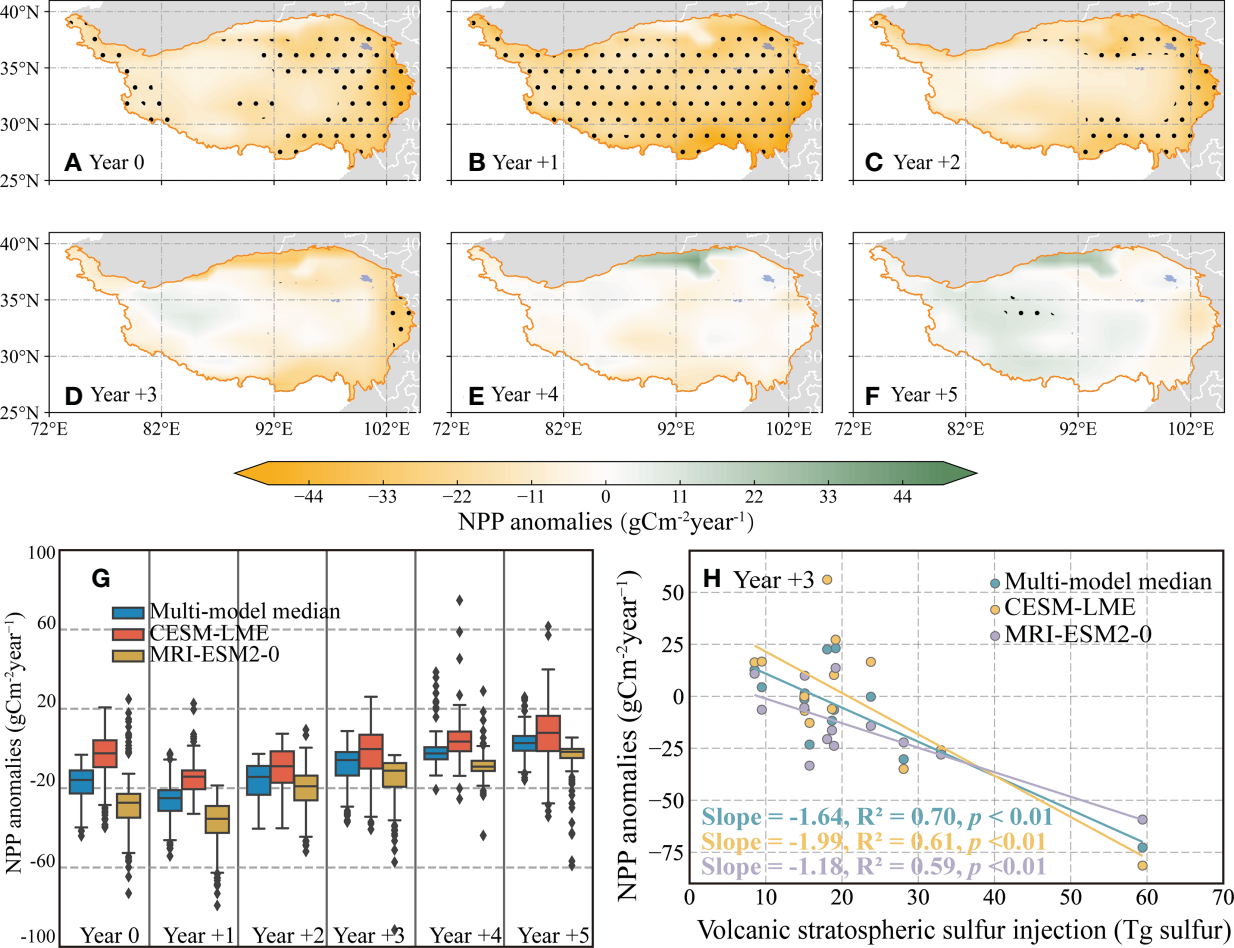
The response of the net primary production (NPP) anomalies to large volcanic eruption events. **(A)** Spatial representation of year 0 the NPP SEA analysis using Multi-model median. **(B–F)** As in **(A)**, but for year +1, year +2, year +3, year +4, and year +5, respectively. The black dot indicates significant regression at the 95% confidence level according to student’s t-test. **(G)** Boxplots showing the spread of the NPP anomalies across the 13 events from 0 years prior to the volcanic event to 5 years following the event, as well as the annual average from Multi-model median (blue), CESM-LME ensemble member 10 (red), and MRI-ESM2-0 (orange). The dark center line in the boxplot characterizes the median, the boxes edges are 25th and 75th percentiles, and the whiskers extend to 1.5 times the median. **(H)** Correlation analysis between the NPP anomalies for the year +3 and the volcanic stratospheric sulfur injection (VSSI). The color lines indicate the linear regressions for the Multi-model median (green), CESM-LME ensemble member 10 (orange), and MRI-ESM2-0 (purple), with the slope value and statistical information shown in the bottom right.

The response of negative NPP anomalies to volcanic eruption events was characterized by a gradual decrease from the eastern (approximately -30 gCm^-2^year^-1^) to the western (approximately 0) in the year 0 and year +2, with the anomalies of the southern and eastern regions are statistically significant at the 95% confidence level (**Figures 2A, C**). Especially, the most significant decrease of NPP occurred in the year +1 across the major of QTP (approximately 94% grid) (**Figure 2B**). Next, the negative NPP anomalies took about 3–5 years to return to normal levels (**Figures 2D, E, F**). We also found that the response of negative NPP anomalies in the CESM-LME and MRI-ESM2-0 models were consistent with the Multi-model median model, while the anomalies of CESM-LME did not respond significantly to volcanic eruption events (**Figures S3**, **S4**).

The NPP on the QTP changed significantly in the year following the volcanic eruption event. Compared to the pre-eruption reference period, the NPP after the volcanic eruption events tended to be smaller, lasting until approximately year +3. Notably, there was largest decrease value occurred in approximately year +1, the medians of negative NPP anomalies came from three models below -14 gCm^-2^year^-1^ (**Figure 2G**). We also noted that negative NPP responses to the large volcanic eruption events were proportional to the estimated magnitude of the eruptions, with a similar negative slope occurring between the year +1 to year +4 (**Figures 2H**, **S2**).

### 3.3 Divergent changes of NPP response across elevation gradients and vegetation types

**Figure 3:**
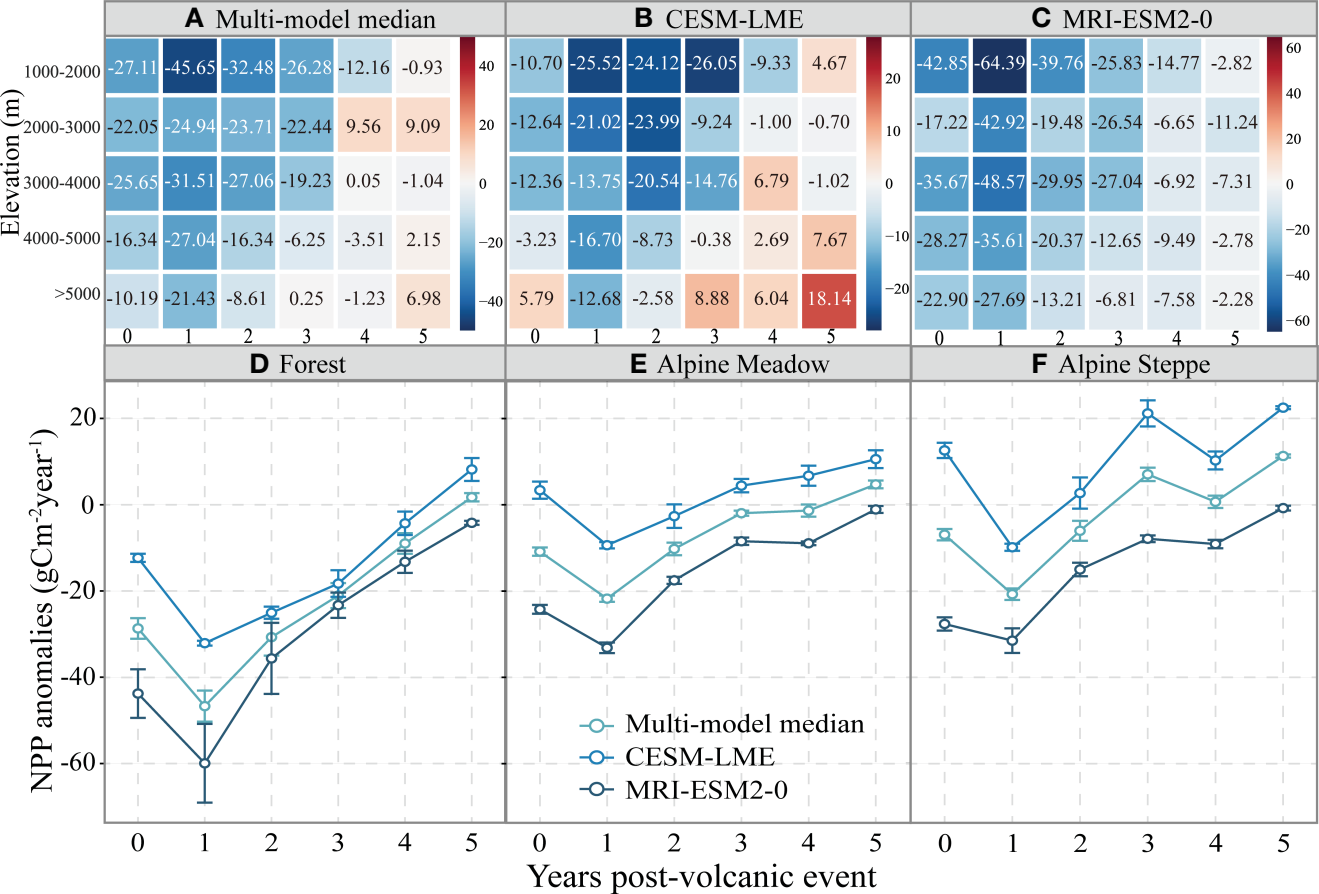
**(A–C)** Heatmap showing regional average responses of the net primary production (NPP) anomalies to the large volcanic eruption events over different elevation gradients from the multi-model median, CESM-LME, and MRI-ESM2-0 model, respectively. The color bars indicate the regional average value of NPP anomalies, unit is gCm-^2^year-^1^. **(D–F)** The responses of the net primary production (NPP) anomalies to the large volcanic eruption events over different vegetation types. The error bars indicate the standard deviation of NPP anomalies over different vegetation types. The circles indicate the regional mean of NPP anomalies.

Overall, elevation played an important role in the response of NPP anomalies to volcanic eruption events (**Figures 3A–C**). The response of negative NPP anomalies to volcanic eruption events decreased with the increase in the average elevation, lasting until approximately year +3. The maximum decrease value of NPP could be observed in the year +1 within the area at an altitude of 1000-2000m (-45.65 gCm^-2^year^-1^ for Multi-model median, -25.52 gCm^-2^year^-1^ for CESM-LME, -64.39 gCm^-2^year^-1^ for MRI-ESM2-0). Moreover, these results demonstrated the NPP anomalies returns to a normal level more quickly at higher elevations (>4000m, approximately 2-3 year) than at lower elevations (<4000m, approximately 3-4 year) after volcanic eruption events.

The response of VNCU to volcanic eruption events varied across vegetation types (**Figures 3D–F**). The negative NPP anomalies for forest (the mean of the multi-model median model is (-46.70 gCm^-2^year^-1^) were greater than the alpine meadow (-21.75 gCm^-2^year^-1^) and alpine steppe (-20.97 gCm^-2^year^-1^) on the QTP in the first year after the eruption. Conversely, the alpine steppe recovered faster than the forest and alpine meadow, and the alpine steppe returning to normal levels within approximately two years, while the alpine meadow recovered in about three years and the forest returned after four years.

### 3.4 Impact of tele-connections forcings on NPP anomalies after volcanic eruptions

**Figure S5** demonstrated these tele-connections forcings (ENSO, AMO, and ITCZ_SA) anomalies to volcanic eruptions. The cooling in the tropical Pacific occurred +4 year after the volcanic eruption events, which was similar to the positional anomaly of ITCZ_SA in response to large volcanic eruption events, but the intensity was weak and barely significant. However, the AMO showed a negative anomaly in the year +1 after the volcanic event, and the negative anomaly of AMO had a long persistence.

**Figure 4:**
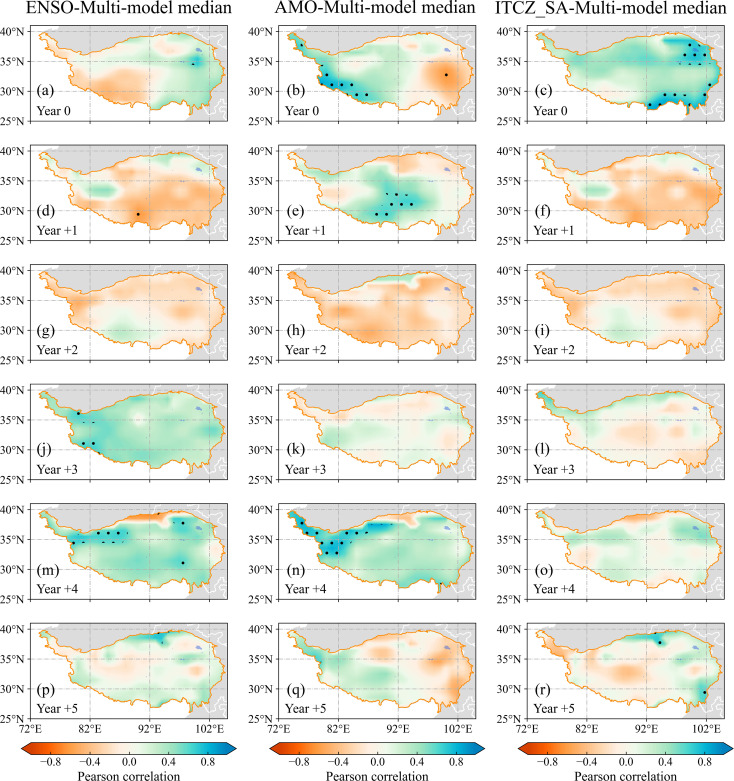
Pearson correlation between **(A, D, G, J, M, P)** ENSO anomaly and the net primary production (NPP) anomalies from Multi-model median model, **(B, E, H, K, N, Q)** AMO anomaly and NPP anomalies, and **(C, F, I, L, O, R)** ITCZ_SA and NPP anomalies across 13 events from 0 years prior of the volcanic event to 5 years following. Black dots show significant correlations at P<0.05 (two-sided Student’s t-test).

The spatial correlations between the anomalies of NPP to the large volcanic eruption events and ENSO, AMO, and ITCZ_SA were shown in **Figure 4**. In the two years following a volcanic eruption, the correlation between NPP anomalies and ENSO indicated a non-significant negative correlation in the major of the QTP (**Figures 4A, D, G**). In contrast, A positive correlation between ENSO and NPP anomalies was observed on the QTP during the year +3 and year +4, with the correlation in the northwestern regions were statistically significant at the 95% confidence level (**Figures 4J, M**), while the signal in MRI-ESM2-0 was barely significant across the QTP (**Figure S7**). For the AMO, the positive correlation between AMO and NPP anomalies was found in year +4 after volcanic eruption events, and the anomalies were statistically significant in the western region (**Figure 4N**). In contrast, the significant positive correlation between AMO and NPP anomalies of the CESM-LME model appeared in the year +2, and the correlation of the year +4 is not significant over the QTP (**Figure S6**). In particular, the positive correlation between ITCZ_SA and NPP anomalies dominated the QTP in year 0 (**Figure 4C**). This stronger VNCU response in the multi-model median was consistent with MRI-ESM2-0 (**Figure S7**).

## 4 Discussion

### 4.1 Assessment of the NPP reconstructed

We examined the performance of the NPP model outputs on the QTP using RMSE, spatial correlation coefficient, and standard deviation. Compare to CESM-LME, the reconstructed NPP of MRI-ESM2-0 have a greater spatial correlation with observations (**Figure 1**). MRI-ESM2-0 with the higher spatial resolution may improve the characterization of mechanisms controlling regional variability and to reduce biases in the mean state (Milinski et al., 2016), while the CESM shows much more moderate multidecadal responses (Tejedor et al., 2021). Although there are uncertainties in the patterns of these paleoclimate models, these models are consistent in the persistence and magnitude of climate anomalies.

### 4.2 Temporal and spatial response of VNCU to the large volcanic eruption

The variation characteristic of NPP anomalies after the large volcanic eruption events implies that VNCU across the QTP was affected by volcanic eruption events, which was in line with earlier studies (Segschneider et al., 2013; Zhang et al., 2019). While the cooling after volcanic eruptions is a consistent response on a global scale (Stoffel et al., 2015; Rao et al., 2017), here we found that the responses of VNCU to volcanic forcing had temporal-spatially heterogeneous under background climate conditions. NPP tended to be smaller after volcanic eruption events compared to the reference period before the eruption, lasting approximately up to +3 years. Notably, the largest decrease values occurred at approximately +1 year (**Figure 2**). As volcanic eruptions cool the land surface, they change the atmospheric circulation resulting in reduced precipitation. It has been shown that monsoonal precipitation in China after volcanic eruptions has been reduced by volcanic aerosols over the past 700 years (Zhuo et al., 2014). Our results also showed that precipitation reduction on the QTP is greatest in the first year after the volcanic eruption (**Figure S8**). Simultaneously, one explanation may be due to the radiative forcing after large volcanic eruptions, which persists for 24 months, determining this anomaly response, lasting until approximately year +3 (Tejedor et al., 2021). These radiative forcings transform the water-heat patterns of the QTP, thus affecting the VNCU (**Figure S6**). Furthermore, the Maximum NPP negative anomalies were primarily distributed in the southeastern, while the low value was mainly in the northwestern (**Figure 3**). Generally, the plant growing season is shorter in the western of the QTP than in the eastern part. Longer winters increase the frequency of freezing and affect water availability (Sun et al., 2022b). Meanwhile, ATP markedly influenced the spatial patterns of NPP anomalies on the QTP under background climate conditions (**Figure S7**). Furthermore, species diversity, the length of the growing season, growth rate, nitrogen content, and photosynthesis gradually increased from northwest to southeast. (Wright et al., 2005; Pan et al., 2009; Yang et al., 2010), these factors may interact with climate anomalies to influence the pattern of VNCU response to large volcanic eruption events. These factors contribute to the understanding of the mechanisms responsible for the lower VNCU in the western part of the QTP compared to the eastern part.

In regard to the response of negative NPP anomalies to the large volcanic eruption events that are influenced by the elevation on the QTP, the negative NPP anomalies decrease with increasing mean elevation. Decreases in leaf area, nodule biomass, stomatal, and mesophyll conductance are more pronounced with increasing altitude (Pan et al., 2009; Frank et al., 2015). These effects weaken the nutrient uptake capacity and photosynthetic of vegetation (Tao et al., 2017; Fei et al., 2018), thus inhibiting the response of VNCU to large volcanic eruption events in high elevation regions.

The VNCU of forests was most sensitive to large volcanic eruption events, followed by alpine meadows and alpine steppe (**Figures 3D–F**). Several factors influence the differences in net carbon uptake in different vegetation types of the QTP. Previous studies indicated that precipitation markedly improves the VNCU, but temperature inhibits it (Sun et al., 2022b). The alpine steppe was mainly found in arid and semi-arid regions. Following volcanic eruptions, precipitation deficits and warmer conditions in the region have combined to increase vapor pressure deficits and soil moisture deficits (**Figures S7**, **S8**) (Frank et al., 2015), and the hotter-drier conditions and asynchronous water-heat relationships have a direct impact on VNCU. Conversely, regarding alpine meadows and forests, foliage area index, photosynthetic enzyme activity, soil phosphorus, and nitrogen content are greater than in alpine steppe, and the better water-heat patterns for several years after volcanic eruptions will strongly promote VNCU (Takashima et al., 2004; Pan et al., 2009). Meanwhile, an appropriate warming condition will promote enzymatic activities in photosynthesis and microbial activity (nitrogen mineralization and soil decomposition rate), which can provide an adequate water-heat environment and nutrient conditions for VNCU (Choat et al., 2018; Sun et al., 2022a). In addition, recovery of VNCU in the alpine steppe to normal levels is shorter than in the alpine meadows and forest. The recovery time of VNCU is mainly influenced by the regional environmental conditions (Gessler et al., 2017; Sun et al., 2022b). Because the unique topography of the QTP obstructs the south Asia monsoon and the westerlies (Ye et al., 2020), Precipitation in alpine steppe (the western region) normalized in the third year after a volcanic eruption, earlier than forest (the southern region) and alpine meadows (the eastern region) (**Figures S7**, **S8**). Simultaneously, the alpine steppe has a simpler functional structure and low productivity than the alpine meadow and forest (Zhuang et al., 2010; Jiang et al., 2014), which may result in shorter recovery times for VNCU. In comparison, alpine meadows and forests are dominated by more complex structures and high productivity, which reflect the recovery ability of VNCU (Stampfli et al., 2018).

### 4.3 Impact of tele-connections forcings on NPP after volcanic eruptions

We focus on the impact of ENSO, AMO, and ITCZ_SA. After volcanic eruptions, Persistent cooling in the North Atlantic (AMO) and tropical Pacific (Niño 3.4) could be observed, which reflected volcanic eruptions will trigger a La Niña-like pattern (**Figure S5**). This result is in line with the previous study (Church et al., 2005; Tejedor et al., 2021). One estimate is that a volcano-induced La Niña-like pattern was most likely to occur in the first year after an eruption, while it was significant until the fourth year after the volcano eruption (**Figure S5**) (McGregor and Timmermann, 2011; Wahl et al., 2014). Furthermore, a positive correlation between ENSO (AMO) and NPP anomalies was observed during the year +3 and year +4 (**Figure 4**). Our results revealed that the effect of volcanic eruptions on the VNCU of QTP was likely to be modulated by moving ENSO and AMO toward their negative phases. While the mechanism remains ambiguous, One explanation may due to changes in SST in the tropics (Rao et al., 2017). Following an eruption, land cools faster than the sea due to the lower heat capacity of the land, and the reduction in surface shortwave radiation. This reduces the thermal contrast between land and sea and weakens the monsoon circulation (Joseph and Zeng, 2011; Rao et al., 2017). In addition, our result indicated a clear positive correlation between ITCZ_SA and NPP anomalies in the year following the eruption (**Figure 4C**). In the Southeast Asia region, the southward movement of the ITCZ causes persistent wetness in this area (Tejedor et al., 2021).

### 4.4 Importance and limitations

Due to the special topography and complex ecosystems, changes in carbon uptake by vegetation on the QTP have attracted considerable interest (Jiang et al., 2014; Sun et al., 2020; Ye et al., 2020). However, the impact of natural variability in VNCU under background climate (i.e., pre-industrial periods) are still poorly understood, especially on millennium time scales. Under the background climate, volcanic eruptions dominate the global climate, which also leads to changes in terrestrial carbon uptake (Toohey and Sigl, 2017; Zhang et al., 2019), whereas the responses of VNCU to the large volcanic eruption events on the terrestrial carbon cycle were not effectively explored. Especially, the recovery and spatial patterns of VNCU after a disturbance were ambiguous. Hence, based on the ESMs, we critically investigated the spatial-temporal patterns of NPP anomalies caused by the large volcanic eruption events on the QTP, and the impact of tele-connections forcings on NPP after volcanic eruptions. As a consequence, these results contribute to our understanding of the effects of volcanic forcing on net carbon uptake by vegetation as well as ecosystems in the pre-industrial period.

In this study, although the volcano dataset is identified to be the most precise reconstruction dataset, in that it still had an average precision of approximately ±2 years (Toohey and Sigl, 2017). As a result, this uncertainty may cause estimates of year zero of volcanic eruption events in SEA analyses to be chosen incorrectly. This uncertainty is not related to the model simulations, but may affect the ESM comparison and can increase or decrease the differences they describe (Tejedor et al., 2021). Moreover, despite previous studies showing differences in spatial patterns, influencing factors, and restoration processes of VNCU depending on climatic conditions and vegetation traits on the QTP, there is still some uncertainty in the magnitude and sign of VNCU (Sun et al., 2022b). Regarding carbon fluxes, there are discrepancies between gross primary production, net ecosystem exchange, total ecosystem respiration, and net primary production (He et al., 2018). Consequently, multiple VNCU datasets are needed to accurately understand the recovery time and dynamics of VNCU.

## 5 Conclusions

We conducted an exhaustive reconstruction of VNCU on the QTP over the last millennium and explored VNCU responses to tropical volcanic forcing. The spatial-temporal pattern and the recovery of VNCU anomalies were primarily driven by climate change and tele-connections forcings after large volcanic eruption events. Notably, environmental conditions and vegetation types also control the variation of VNCU. Under the background climate, the VNCU of the QTP tended to decrease after volcanic eruption events compared to the reference period before the eruption, lasting approximately up to +3 years, and the largest decrease values occur at approximately +1 year. The spatial patterns and temporal dynamics were mainly driven by climate change and modulated by predisposing ENSO and AMO toward their negative phases. Furthermore, elevation was also an undeniable and essential driving force associated with VNCU on the QTP. The magnitude of NPP anomalies after volcanic eruptions decreased with increasing elevation. Next, the divergence of different vegetation types on the QTP to the anomaly responses and recovery process of the VNCU after volcanic eruption was prominent. This is attributed to the impact of different water-heat patterns and vegetation types. Therefore, our model-based analysis emphasized the importance of further investigating the VNCU responses to tropical volcanic forcing and devoting more attention to the mechanisms of VNCU in unique geographical regions and vegetation types.

## Data availability statement

The original contributions presented in the study are included in the article/**Supplementary Material**. Further inquiries can be directed to the corresponding author.

## Author contributions

ZY and ZW designed research. JX and ZW collected the data. ZY analyzed data. ZY and JT wrote the manuscript. ZY and ZW participated in the reviewing of methodology, results, and manuscript. All authors contributed to the article and approved the submitted version.
