# Supplementary material for: Tropical volcanic eruptions reduce vegetation net carbon uptake on the Qinghai–Tibet Plateau under background climate conditions
Source: Front Plant Sci. 2023 Mar 16;14:1122959. doi: 10.3389/fpls.2023.1122959 (PMC10061027; doi:10.3389/fpls.2023.1122959)
Supplement: Supplementary file 1 [file DataSheet_1.docx]

Supplementary Material

# Supplementary Text, Figures and Tables

The supporting information contains additional text for the methods section, the seven supporting figures mentioned in the main text, and one supporting table referenced in the main text.

## Supplementary Text

Text S1. MRI-ESM2-0 and CESM-LME models output and data processing

We use ESM diagnostics of monthly precipitation (pr in MRI-ESM2-0, and PRECT in CESM-LME), near-surface (usually, 2 meter) air temperature (tas in MRI-ESM2-0, and TS in CESM-LME). Only one ensemble member per model is included (r1i1p1). For all variables, the original values are smoothed with an 11-year moving window. To summarize the multi-model ensemble statistics, we employ bilinear interpolation to regrid the CESM-LME and MRI-ESM2-0 model to a common 1°×1° grid (Schulzweida et al., 2006), and then take the multi-model median for each grid cell (Li et al., 2021). In this configuration, we conduct an exhaustive reconstruction of VNCU on the QTP over the last millennium and explore VNCU responses to tropical volcanic forcing.

Text S2. Divergent changes of NPP response in different vegetation types and elevation

The vegetation distribution map of the QTP is obtained from the 1:1000000 Chinese vegetation map in the Resource and Environment Data Cloud Platform. and the spatial patterns of the main vegetation types (alpine meadows, alpine grasslands, and forests) on QTP are shown in Figure S1. To ensure the robustness of the results, we selected typical areas of vegetation types on the QTP. These typical vegetation type areas consisted of four 1° × 1° grids, and every area contained only one vegetation type. Under this limitation, we reflected the effect of vegetation type on VNCU variation by assessing the variation of VNCU in the vegetation type areas. Digital elevation model (DEM) product is acquired from Geospatial Data Cloud. the spatial resolution of DEM is approximately 90m×90m grid.

Text S3. Effect of volcanic eruption intensity on net carbon uptake by vegetation

Although our selection process is consistent with the state-of-the-art selection procedures in the literature, all eruptions are different, and estimates of eruption volume do not imply the proportion of aerosols deposited in the stratosphere, nor does the composition of the sediment vary with each eruption (Tejedor et al., 2021). The link between the VNCU response of an eruption and the estimated eruption size remains relatively ambiguous. Therefore, we analyzed the link between the changes in VNCU and eruption volume in different years after the eruption.

## Supplementary Figures

**
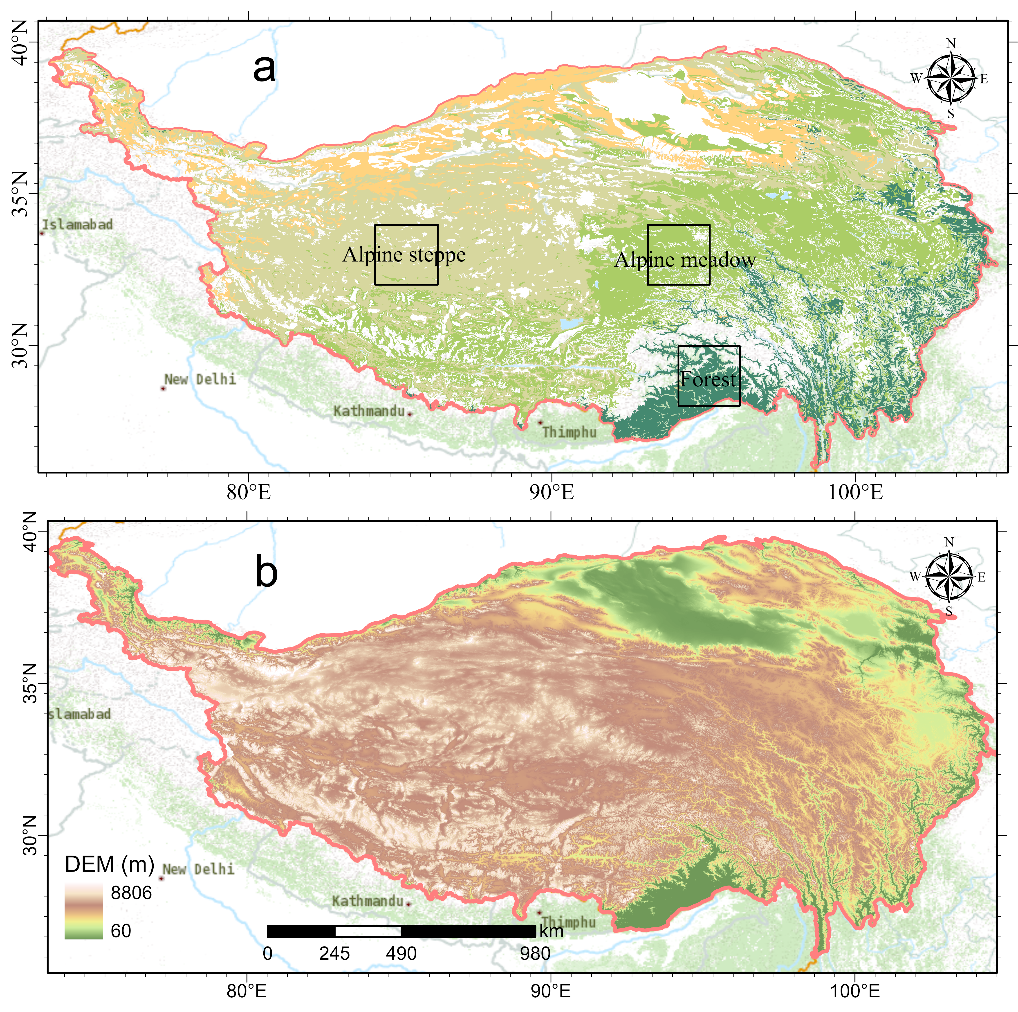
**

Figure S1. Type or paste caption here. Geographical information on the QTP, including the border, vegetation types (forest, alpine desert, steppe and meadow), and elevation.


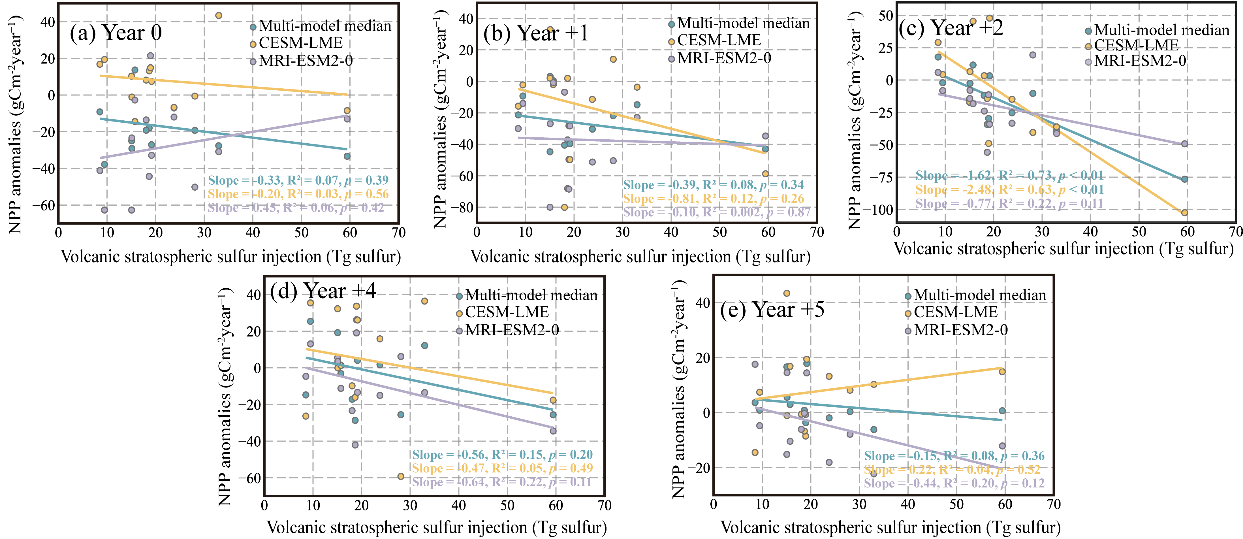


Figure S2. Correlation analysis between the NPP anomalies and the volcanic stratospheric sulfur injection (VSSI) for the year 0, +2, +3, +4, +5. The color lines indicate the linear regressions for Multi-model median (green), CESM-LME ensemble member 10 (orange), and MRI-ESM2-0 (purple).


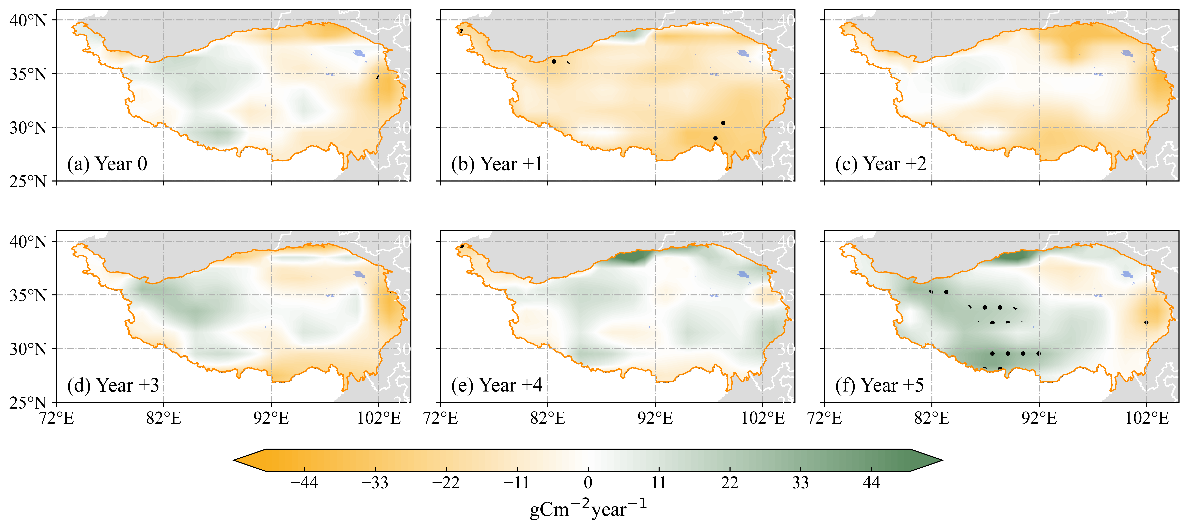


Figure S3. The spatial response of the net primary production (NPP) anomalies to last millennium large volcanic eruption events in CESM-LME. (a) Spatial representation of year 0 the NPP SEA analysis using CESM-LME. (b-f) As in (a), but for year +1, year +2, year +3, year +4, year +5, respectively. The black dot indicates significant regression at the 95% confidence level according to Student's t-test.


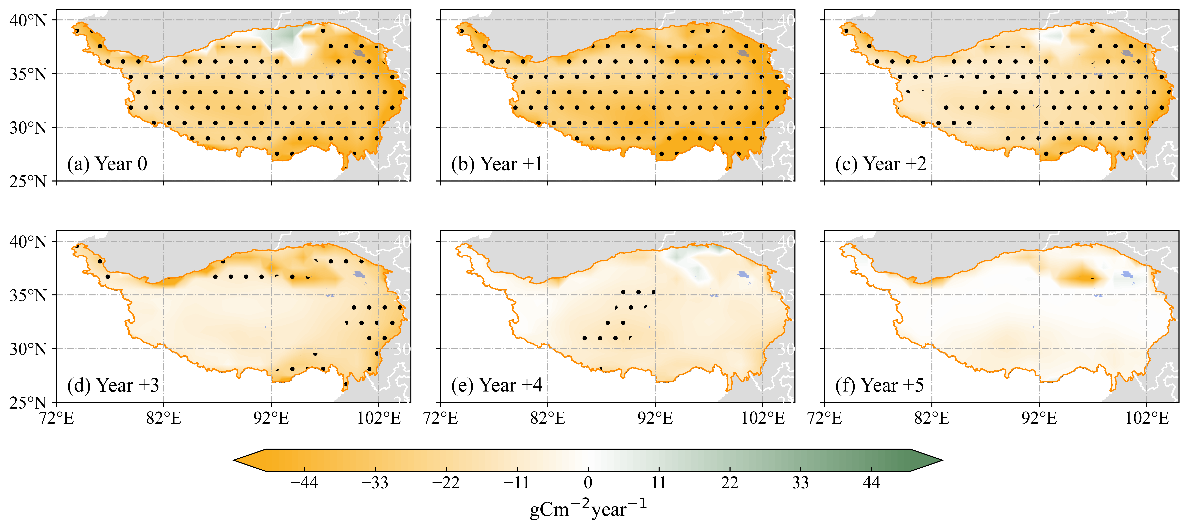


Figure S4. The spatial response of the net primary production (NPP) anomalies to last millennium large volcanic eruption events in MRI-ESM2-0. (a) Spatial representation of year 0 the NPP SEA analysis using MRI-ESM2-0. (b-f) As in (a), but for year +1, year +2, year +3, year +4, year +5, respectively. The black dot indicates significant regression at the 95% confidence level according to Student's t-test.

**
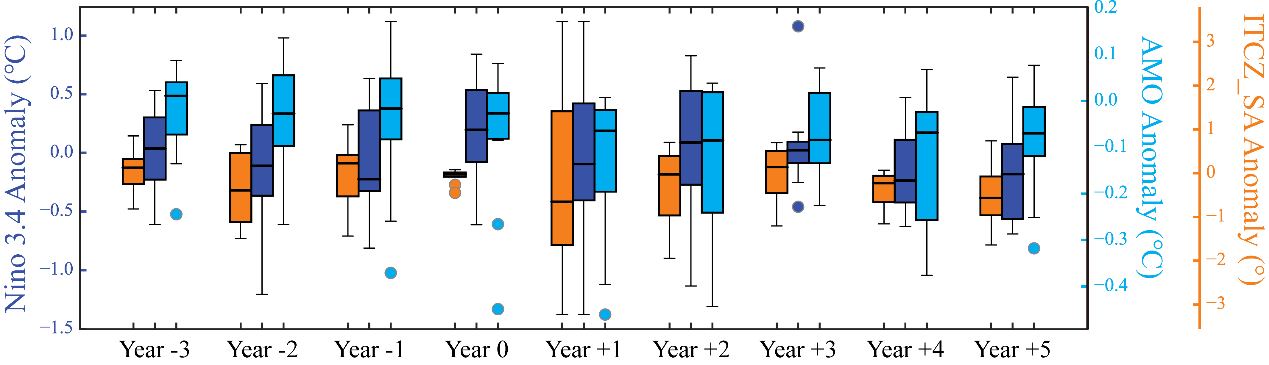
**

Figure S5. Boxplot shows that the response of three tele-connections forcings (ENSO, AMO, and ITCZ_SA come from PHYDA) anomalies to the large volcanic eruption events across the 13 volcanic eruption events from years -3 prior to years +5 following the event. as well as the anomaly from Niño 3.4 indices (deep blue), AMO indices (light blue), and the location (in the degree of latitude) of South Asia ITCZ (orange). The dark center line in the boxplot represents the median, the edges of the boxes are 25th and 75th percentiles, and the whiskers extend to 1.5 times the median.


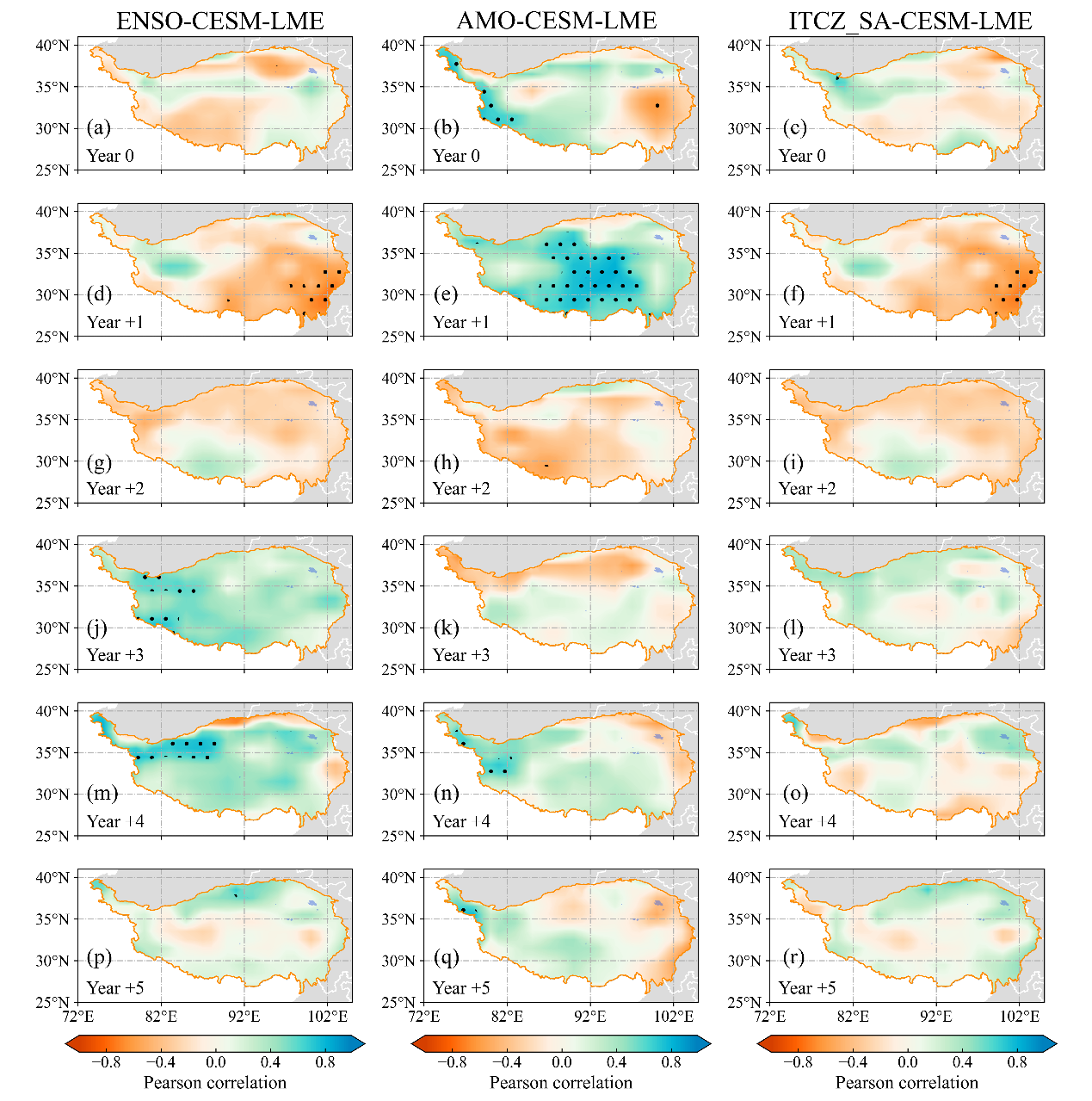


Figure S6. Same as in figure 7, but for the CESM-LME model.


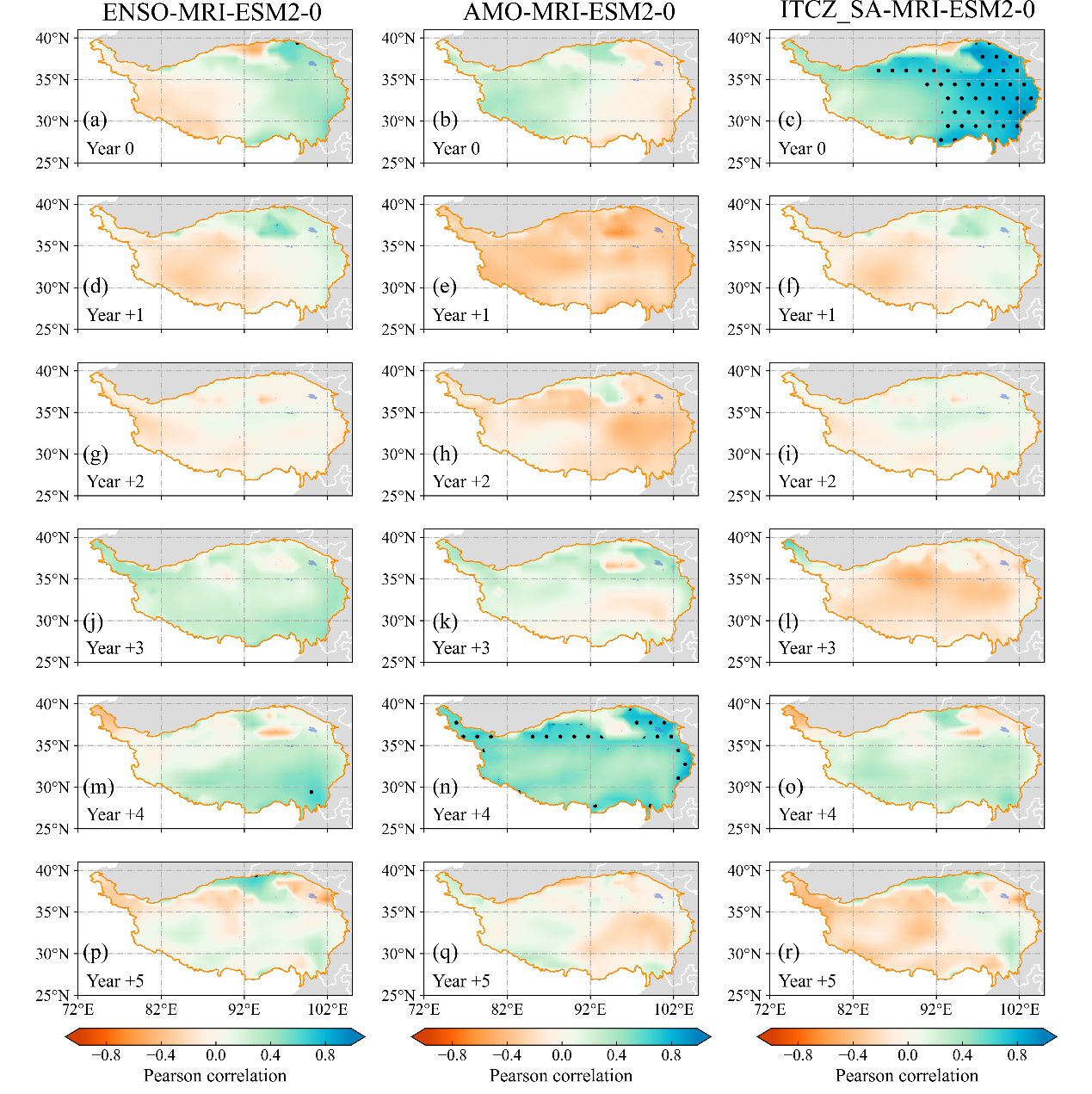


Figure S7. Same as in figure 7, but for the MRI-ESM2-0 model.


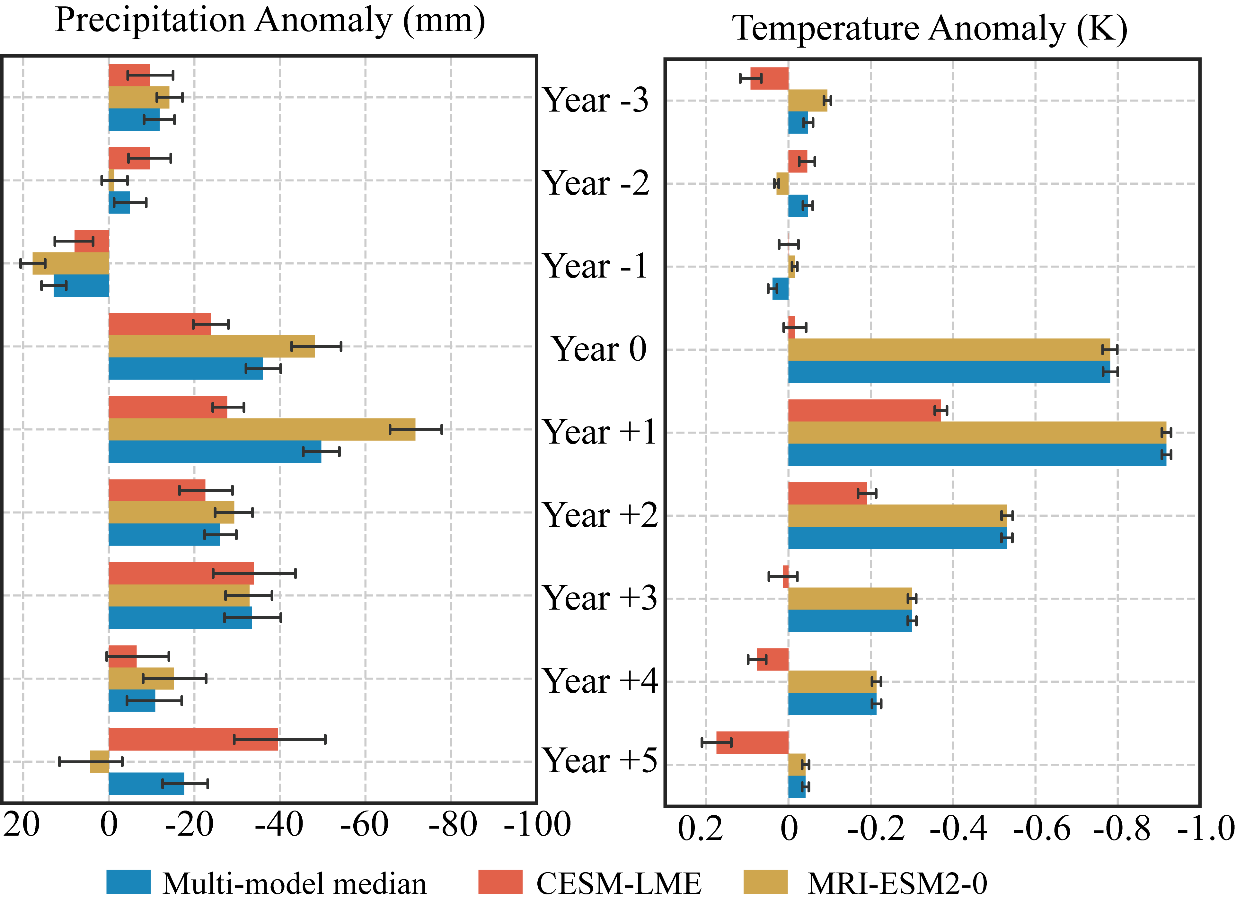


Figure S8. Precipitation anomaly (left column) and temperature anomalies (right column) across 13 events from 0 years prior of the volcanic event to 5 years following. Error bars represent standard deviation.


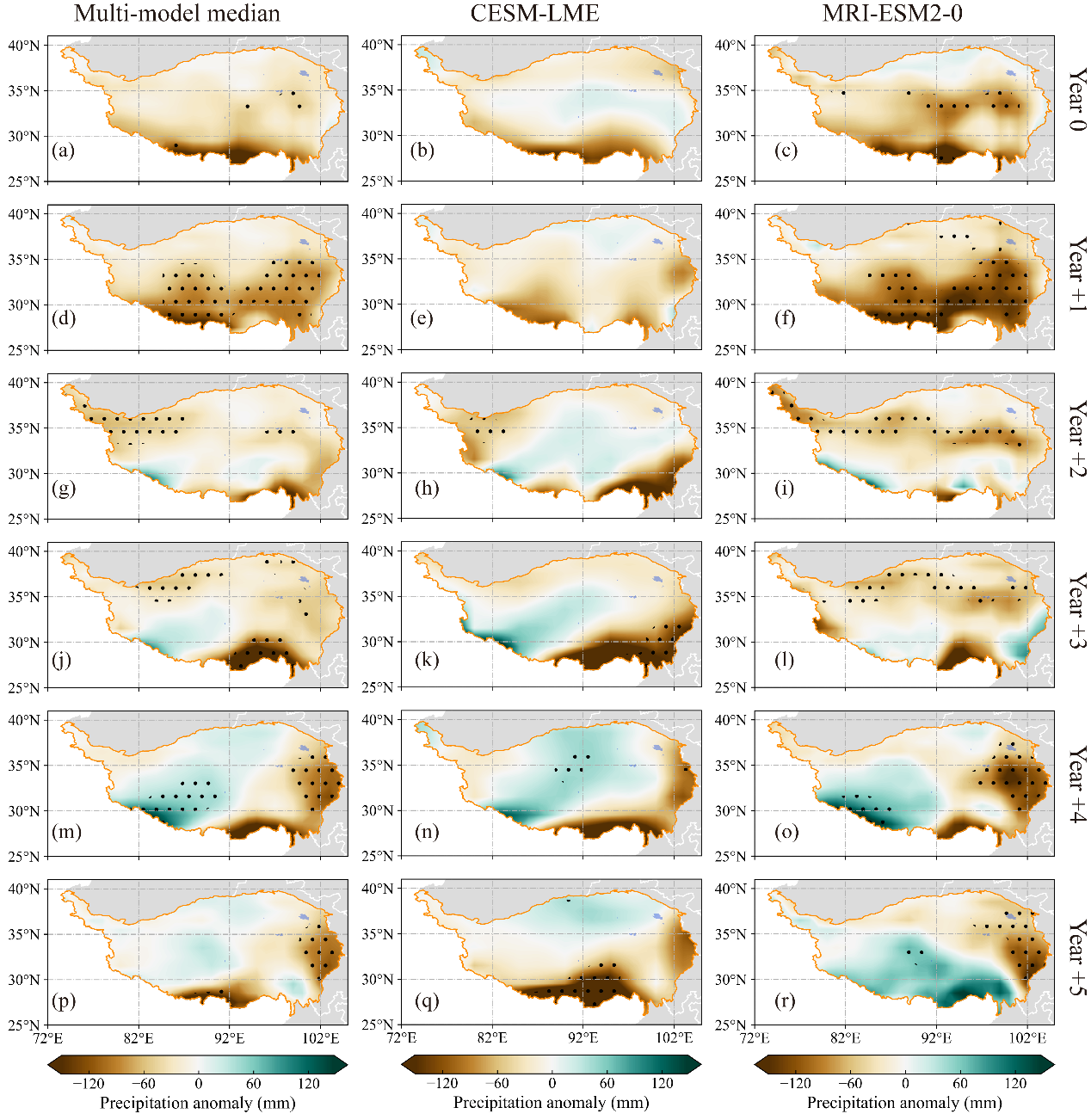


Figure S9. The spatial response of the precipitation anomalies to last millennium large volcanic eruption events in Multi-model median model (left column), CESM-LME (middle column), MRI-ESM2-0 (right column). The black dot indicates significant regression at the 95% confidence level according to Student's t-test.


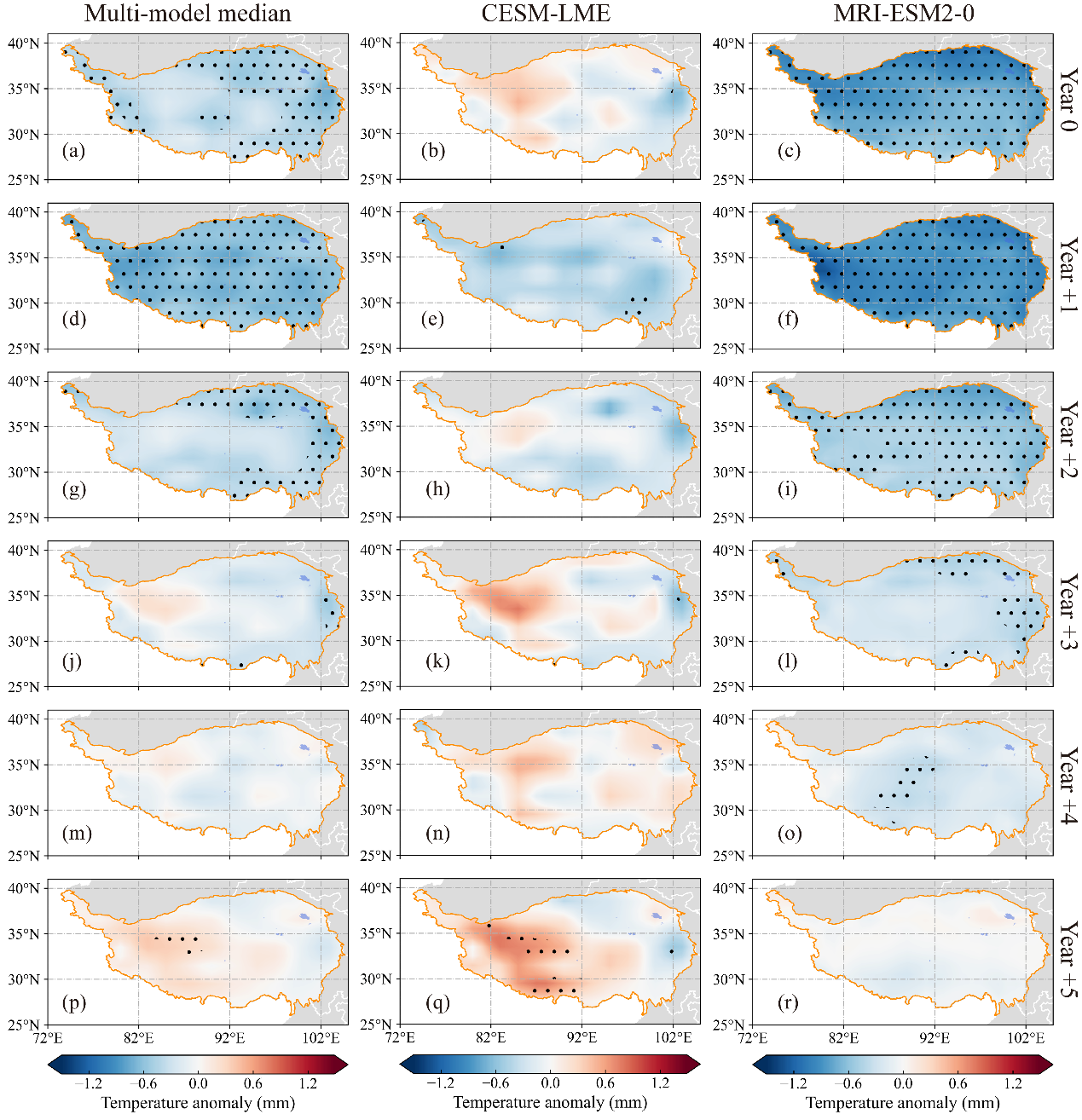


Figure S10. The spatial response of the temperature anomalies to last millennium large volcanic eruption events in Multi-model median model (left column), CESM-LME (middle column), MRI-ESM2-0 (right column). The black dot indicates significant regression at the 95% confidence level according to Student's t-test.

Table S1. Detailed descriptions of 13 selected volcanic eruption events

| Year (CE) | Latitude | VSSI (Tg [S]) |
| --- | --- | --- |
| 1108 | 0 | 19.16 |
| 1171 | 0 | 18.05 |
| 1191 | 0 | 8.53 |
| 1230 | 0 | 23.78 |
| 1257 | -8.4 | 59.42 |
| 1286 | 0 | 15.06 |
| 1345 | 0 | 15.11 |
| 1458 | 0 | 32.98 |
| 1600 | -16.6 | 18.95 |
| 1640 | 6.1 | 18.68 |
| 1695 | 0 | 15.74 |
| 1815 | -8 | 28.08 |
| 1835 | 13 | 9.48 |

**References**

Li, C., Zwiers, F., Zhang, X., Li, G., Sun, Y., Wehner, M., (2021). Changes in annual extremes of daily temperature and precipitation in CMIP6 models. Journal of Climate 34, 3441-3460.

Schulzweida, U., Kornblueh, L., Quast, R., (2006). CDO user’s guide. Climate data operators 1, 205-209.

Tejedor, E., Steiger, N.J., Smerdon, J.E., Serrano-Notivoli, R., Vuille, M., (2021). Global hydroclimatic response to tropical volcanic eruptions over the last millennium. Proceedings of the National Academy of Sciences 118, e2019145118.
